# A hybrid compound scaling hypergraph neural network for robust cervical cancer subtype classification using whole slide cytology images

**DOI:** 10.1038/s41598-025-05891-4

**Published:** 2025-07-01

**Authors:** Pooja Govindaraj, Sasikaladevi Natarajan, Pradeepa Sampath, Akilesh Thimma Suresh, Rengarajan Amirtharajan

**Affiliations:** 1 Department of Computer Science and Engineering, School of Computing, Shanmugha Arts Science Technology and Research Academy, Thanjavur, Tamil Nadu 613401 India; 2 Department of Information Technology, School of Computing, Shanmugha Arts Science Technology and Research Academy, Thanjavur, Tamil Nadu 613401 India; 3 Department of Electronics and Communication Engineering, School of Electrical & Electronics Engineering, SASTRA Deemed University, Thanjavur, Tamil Nadu 613401 India

**Keywords:** Cervical cancer, Compound scaling convolutional neural network, K-dimensional hypergraph neural network, Whole slide cytology image, Propagation, Mathematics and computing, Information technology

## Abstract

Cervical cancer is a major cause of mortality among women, particularly in low-income countries with insufficient screening programs. Manual cytological examination is time-consuming, error-prone and subject to inter-observer variability. Automated and robust classification of the whole slide cytology images for cervical cancer is essential for detecting precancerous and malignant lesions. We propose a novel deep learning framework, the Compound Scaling Hypergraph Neural Network model (CSHG-CervixNet), for robust classification of cervical cancer subtypes. The model integrates a Compound Scaling Convolutional Neural Network (CSCNN) with a k-dimensional Hypergraph Neural Network (kd-HGNN) architecture. CSCNN balances the network’s depth, width, and resolution, supporting effective feature representation with minimal computational overhead. kd-HGNN captures higher-order relationships between the features, and its propagation mechanism ensures better feature diffusion across distant nodes. The model is evaluated on the benchmark Sipakmed dataset and achieves an accuracy of 99.31%, with a macro-averaged precision of 98.97%, recall of 99.38%, and F1-score of 99.34%, demonstrating its robustness in cervical cancer subtype classification. Pathologists and other medical experts will find this study helpful in distinguishing cervical cancer subtypes so that targeted treatment may be provided and effective disease management is made possible.

## Introduction

Cervical Cancer (CC) ranks as the fourth most prevalent cancer affecting women globally and accounts for a significant portion of cancer-related deaths^1–4^. According to recent statistics in 2020, it is estimated that Cervical Cancer caused over 600,000 new cases and about 340,000 deaths^5–7^. This projection suggests that by 2030, Cervical cancer-related deaths could escalate to 400,000 people annually. According to CC cytology, the subtypes are commonly categorised into five distinct subtypes: Metaplastic, Dyskeratotic, Koilocytotic, Superficial-Intermediate and Parabasal^8–10^. Accurate recognition and classification of these subtypes are crucial for cancer diagnosis and personalised treatments^11^.

Traditionally, Cervical Cancer subtyping relies on manual analysis of WSIs by expert pathologists, which is error-prone, subjective and labour-intensive^9,12,13^. Subtypes tend to have visual variations, which makes it challenging even for skilled pathologists to classify them. Furthermore, staining artifacts, overlapping cell features, and intra-class variations also make it more difficult for the classification task, which has the potential to cause diagnostic inaccuracies^14^. Therefore, an automated Computer-Aided Diagnostic (CAD) tool to classify cervical cancer subtypes from WSIs is essential for improving accuracy, reducing workload, and facilitating early and precise treatment planning^15,16^.

Many deep-learning techniques have shown considerable promise in detecting various cancers, such as lung, breast, cervical, and colorectal^17–19^. In recent studies, Convolutional Neural Networks (CNNs) are the basis of several CAD systems for evaluating cervical cytology images, significantly increasing diagnostic efficiency and precision^20–24^. However, CNNs effectively extract discriminative features; they often struggle to capture complex, multi-relational interactions among features^25–28^. Transformer-based architecture has recently gained attention for its powerful self-attention mechanisms in modelling global contexts, especially for medical image applications^29,30^. They are associated with drawbacks such as model complexity, large data requirements, and lack of interpretability in resource-poor clinical environments. Even though they have demonstrated promising results, they likely lack fine-grained, higher-order feature modelling capabilities, which are crucial for tasks such as cytological subtype classification.

To address the limitations of existing approaches, recent work has explored Graph Convolutional Networks (GCN), which have sought to reduce the inherent shortfalls of traditional approaches by adopting pairwise interactions^31–33^. However, standard GCNs are restricted from operating on pairwise (2-node) connections, whereas Hypergraph Neural Networks (HGNNs) have gained increased attention by enabling multi-way relational modelling through hyperedges. However, many conventional HGNN structures still use static structures and cannot handle higher-order correlations in large, heterogeneous datasets^25,31–34^. A hybrid subtype classification framework, Compound Scaling Hypergraph neural network (CSHG-CervixNet), is proposed to overcome these issues. The model utilises efficient Compound Scaling Convolutional Neural Network (CSCNN) deep feature extraction and modified k-dimensional Hypergraph Neural Network (kd-HGNN) for robust classification. We adopt CSCNN as a backbone for feature extraction, which uniformly scales depth, width and resolution, enabling the model to extract deep, multi-scale features from whole slide cytology images. The kd-HGNN models higher-order relationships of the extracted features through hypergraph construction based on neighbourhood similarity, ensuring comprehensive feature aggregation and precise classification. By embedding a feature propagation mechanism in the hypergraph architecture, we effectively model the diffusion of feature information between local and global relational contexts to capture inter-cellular dependencies inherent in cytological images more accurately. Leveraging the publicly available SipakMed dataset, our model is meticulously designed to address the nuanced morphological variances across subtypes, ensuring accuracy and scalability.

The major contributions of this study are threefold:

- Compound Scaling Convolutional Neural Network for deep feature extraction: CSCNN is utilised to extract the deep features from cervical whole slide cytology images. The uniform scaling of depth, width and resolution ensures an efficient feature extraction process, capturing intricate morphological variations crucial for subtype classification.
- Development of K-dimensional Hypergraph Neural Network for classification: A robust kd-HGNN architecture that effectively models higher-order relationships among the extracted feature vectors. The hyperedges are based on neighbourhood similarity. The kd-HGNN facilitates comprehensive feature aggregation, thereby enhancing classification accuracy.
- A comprehensive evaluation of the SipakMed dataset: Extensive experiments are conducted on the benchmark SipakMed cervical cytology dataset. The results demonstrate that the proposed hybrid framework outperforms the baseline methods in cervical cancer subtype classification tasks.

The article is structured as follows: “Related works” explains the detailed literature review based on Machine Learning, Deep Learning, and hybrid techniques. “Proposed CSHG-CervixNet architecture” introduces the proposed methodology. “Experimental analysis and discussion” presents the experimental results and discussion. “Conclusion” summarises the conclusion and future work.

## Related works

Machine Learning (ML) and Deep Learning (DL) techniques have significantly advanced cervical cancer diagnosis. These techniques have been used to improve automated diagnostic models’ accuracy, dependability, and interpretability while addressing issues including cytological image variability, the requirement for strong feature extraction and robust classification. This section examines the existing studies in three main areas: (1) ML approaches, which prioritise feature engineering and conventional classification algorithms; (2) DL methods, which employ CNNs and complex architectures to achieve increased accuracy and generalizability; and (3) ensemble and hybrid models, which integrate various approaches to enhance performance and robustness. This review contextualises and illustrates the development of techniques by evaluating various approaches.

### Deep learning-based frameworks

A ResNet-based Autoencoder^35^ was proposed for cervical cell classification by using an attention mechanism achieving an accuracy of 99.26%. A cervical pap smear image classification model named CervixFormer^36^ which utilises the Swin Transformer achieved an accuracy of 98.29%. Muksimova et al.^37^ proposed a Reinforcement Learning (RL) based ResNet-50 that utilises supporter blocks to highlight essential feature information and a meta-learning ensemble to improve segmentation accuracy. Another model, CerviLearnNet^38^ automates cervical cancer diagnosis by combining RL with a modified Efficient-NetV2 model. A CNN with four convolutional layers^39^ was used to categorise cervical cells into five groups using the SipakMed dataset, with an accuracy of 91.13%. Bhatt et al.^40^ proposed a convolutional-based cervical pap smear image classification model utilising a progressive resizing technique that demonstrated an accuracy of 99.70%. Lin et al.^41^ employed a CNN architecture that had already been trained to extract essential features of CC pap smear images. These features are classified by using an SVM classifier, achieving an accuracy of 94.5%. Rehman et al.^42^ believe that transfer learning is a useful technique for resolving the issues of overfitting and excessive parameter correlation. DenseNet121 was used by Chen et al.^43^ to improve the classification rate of lightweight CNNs for cervical cell categorisation, and they achieved a classification accuracy of 96.79%. Mohammed et al.^44^ utilised a pre-trained DenseNet169 and attained a classification accuracy of 99% for five-class cervical pap smear images. Using ViT-CNN and CNN-LSTM, Maurya et al.^45^ introduced a computer-aided diagnostic system for classifying cervical cell subtypes. The model attained an accuracy of 97.65%. Combining the features from the visual transformer and pre-trained DenseNet201, as utilised by Hemalatha et al.^46^ extracted both local and global features of cervical cell images. Based on these combined features, fuzzy feature selection was then used. The model attained an accuracy of 98.13%. Attallah^47^ proposed the CerCanNet model, which integrates ResNet18 and Quadratic Support Vector Machine (QSVM) for pap smear cervical image classification, which attains an accuracy of 96.3% for the SipakMed dataset.

### Machine learning-based models

Several ML-based approaches have been explored for cervical cancer diagnosis. A recent approach for CC diagnosis using the Gazelle Optimisation Algorithm (GOA) was proposed by Nour et al.^48^ It uses an improved MobileNetv3 architecture for feature extraction, and a Stacked Extreme Learning Machine (SELM) was employed for classification. A two-phase classification model^49^ based on the HErlev dataset was proposed, achieving 98.80% accuracy. The approach involved extracting texture features from nucleolus and cytoplasm, then classification through an optimised multilayer feed-forward neural network. DenseNet169, a technique that combines RCNN architecture with an attention pyramid network, was employed by Cao et al.^50^ and the model attained an accuracy of 95.08%. Medical experts had to annotate the labels manually and bounding boxes using this procedure, which was time-consuming. CerviXpert, proposed by Akash et al.^51^ utilises deep CNN for cervical pap smear image subtype classification, which achieved accuracies of 98.04% and 98.60% for three-class and five-class classifications, respectively. Liu et al.^20^ proposed a CVM-Cervix framework for cervical pap smear image classification, which attained an accuracy of 92.87%. The model combines Xception and Multilayer Perceptron (MLP) for classification. Integrating a Stacked Autoencoder with Generative Adversarial Networks (SOD-GAN) has been explored to facilitate lesion detection and classify cervical cell images into premalignant and malignant categories. Another study^52^ addressed segmentation and feature extraction issues with an accuracy of 97.08%. To enhance the accuracy of the cervical cancer prediction model, Ijaz et al.^53^ used outlier removal techniques such as DBSCAN and iForest. Graph Convolution Network (GCN) was utilised by Shi et al.^54^ for cervical pap smear classification, which attained an accuracy of 98.37%.

### Hybrid models

Hybrid and ensemble models are becoming more popular for diagnosing cervical cancer, cancer grading and subtype classification. The CompactVGG model in^55^ demonstrated classification accuracies of 97.80% and 94.81%, respectively, on the HErlev and SipakMed datasets. Huang et al.^56^ utilised DenseNet-121, VGG-16, ResNet-50, and Inception v3 on various datasets, including HErlev and SipakMed. The highest accuracy was achieved by DenseNet-121 95.33%. Dong et al.^5^ proposed a hybrid model called BiNext-Cervix for cervical cancer subtyping. The model combines ConvNext and BiFormer and achieves an accuracy of 83.51%. Chauhan et al.^57^ utilised the progressive resizing technique with Principal Component Analysis (PCA) to classify cervical pap smear images and attained an accuracy of 98.97% for five-class classification and 99.29% for 2-class classification. Wu et al.^58^ employed a combination of two models CNN and transformers, named the CTCNet model, which attained an accuracy of 97.74%. Other studies explored object identification algorithms, including CenterNet, Faster R-CNN, and YOLOv5 by Xu et al.^59^ for cervical cancer detection. The aforementioned model’s tolerance to high variability and scalability to larger data sets remains challenging. Chauhan et al.^60^ utilised a hybrid-based network for cervical whole slide image classification and attained an accuracy of 97.45% and 99.49% for the sipakmed and LBP datasets, respectively. Table 1 summarises various state-of-the-art cervical cancer subtype classification techniques using pap smear images.

Earlier works have explored graph-based models in medical image analysis, but these models rely mostly on simple graph constructions and traditional CNNs. In contrast, our proposed architecture introduces a CSCNN to perform effective deep feature extraction combined with a k-dimensional hypergraph model to capture higher-order, non-pairwise relationships of features. With this, more complicated and semantic interactions can be simulated.

**Table 1:** Summary of important existing models for CC subtype classification.

Method	Description	Datasets	Accuracy
BiNext-Cervix^5^	ConvNext and BiFormer models	SipakMed	83.51%
Progressive Resizing approach + PCA^57^	Extracts features using ResNet-152 and VGG-16 with progressive resizing (224 × 224 → 1024 × 1024); PCA for dimensionality reduction + Majority voting based classification (SVM + RF)	SipakMed + LBC	98.47%
CTCNet^58^	CNNs and Transformers. Deformable Large Kernel Attention (DLKAttention)	SipakMed	97.74%
6 Deep learning models + SVM^61^	VGG16, Xception, DenseNet169, InceptionV3, ResNet101, and Inception ResNet + SVM for classification	SipakMed	95.66%
Improved CervicalNet^62^	U-Net for segmentation + GCN for classification	SipakMed + Herlev	Accuracy-98.61% precision-97.33%, specificity-97.12%, recall- 97.11%, F1-score-97.56%
MaxCerVixT^63^	CNN-based ViT	SipakMed	99.02%
TL based CNN^40^	TL based EfficientNet B3 and progressive resizing	SIPaKMed	99.70%
CervixFormer^36^	Swin Transformer	SipakMed, and Cervix93	SipakMed-98.29% Cervix93-97.01%
VisionCervix^45^	Vision Transformer (ViT) and fine-tuned MobileNet	SipakMed	Accuracy- 97.65%, precision-99.54%, recall- 97.65%, f1 score- 98.58%
CVM-Cervix^20^	Xception model, ViT, MLP	CRIC, SipakMed	Accuracy- 92.87%, precision-92.80%, recall- 92.90%, f1 score- 92.80%
CNN^39^	CNN	SipakMed	Accuracy-91.13%
CACCD-GOADL^48^	MobileNetv3 model with Gazelle Optimizer Algorithm (GOA)	Herlev	f1 score- 95.71%, Accuracy- 98.69%, recall- 95.37%, precision-95.24%
CytoBrain^55^	CompactVGG	Cervical cancer WSI images	Accuracy-88.30%, specificity-91.03%, f1 score-87.04%, Sensitivity-92.83
SOD-GAN + F-SAE^52^	Fine-tuned Stacked Autoencoder based GAN	Colonoscopy images	Accuracy-94.8%
GCN^54^	Graph Convolution Network	SipakMed	Accuracy-98.37%
CerCanNet^47^	ResNet18 + Quadratic Support Vector Machine	SipakMed	Accuracy-96.3%
CerviXpert^51^	Customised CNN	SipakMed	Accuracy for three class classification-98.04%, Five class classification- 98.60%

### Proposed CSHG-CervixNet architecture

**Fig. 1:**
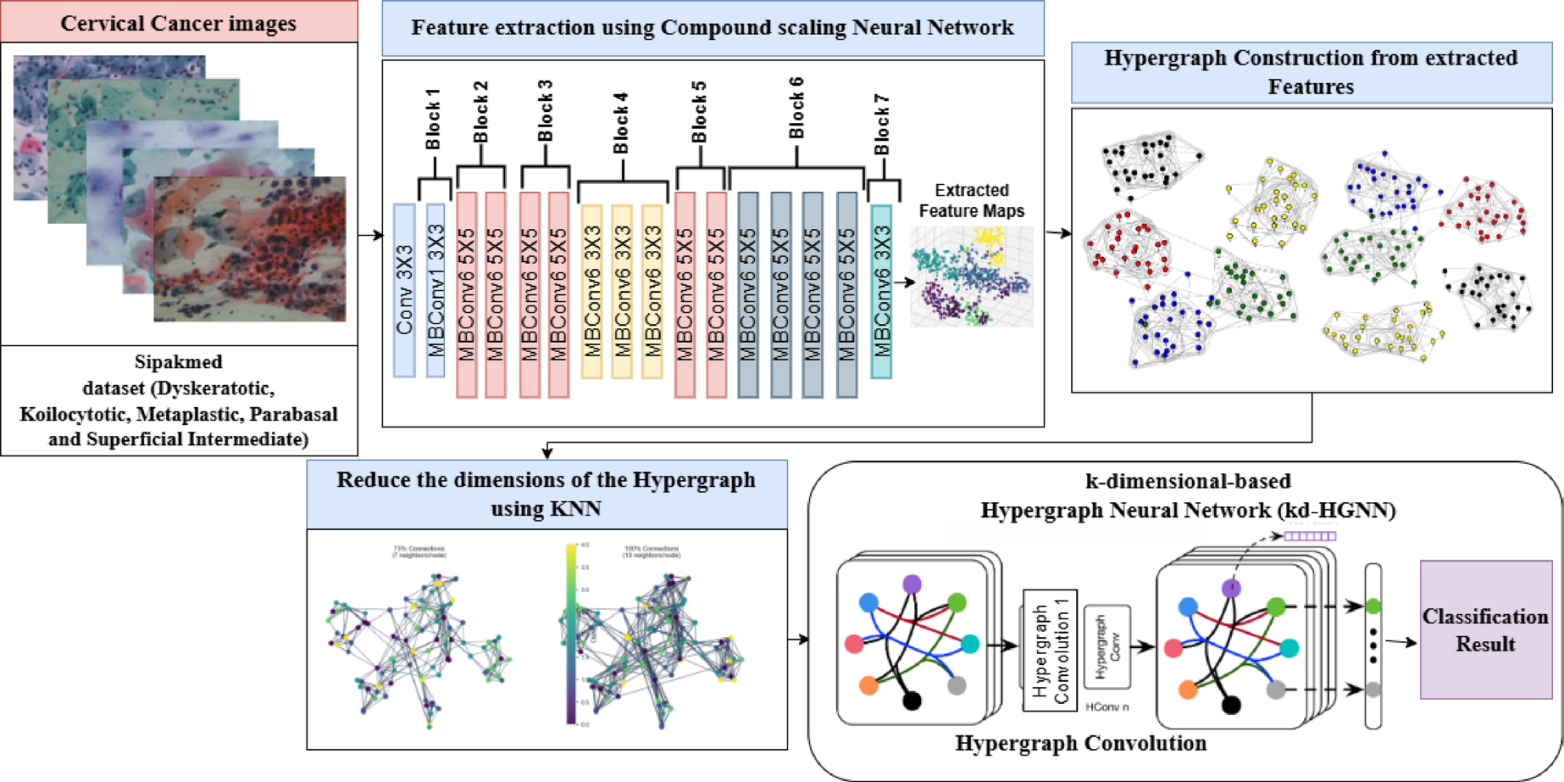
CSHG-CervixNet architecture.

Figure 1 illustrates the overall working methodology diagram. The proposed hybrid model CSHG-CervixNet is trained and validated using the SipakMed cervical cancer cytology images. The model integrates a Compound Scaling Convolutional Neural Network (CSCNN) for feature extraction and a k-dimensional-based Hypergraph Neural Network (kd-HGNN) for robust classification.

### Data set description

**Fig. 2:**
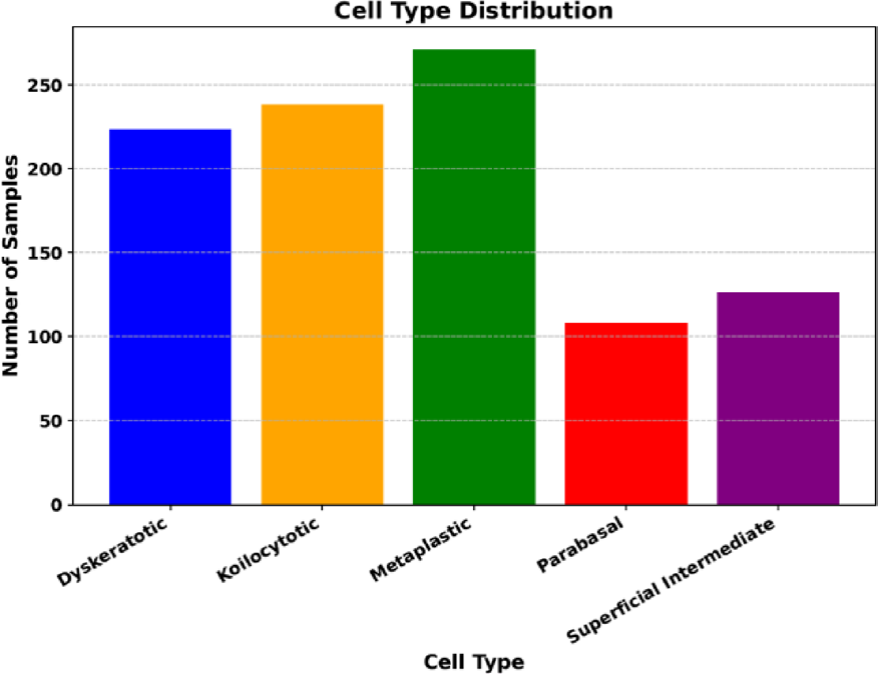
Cell type distribution.

**Fig. 3:**
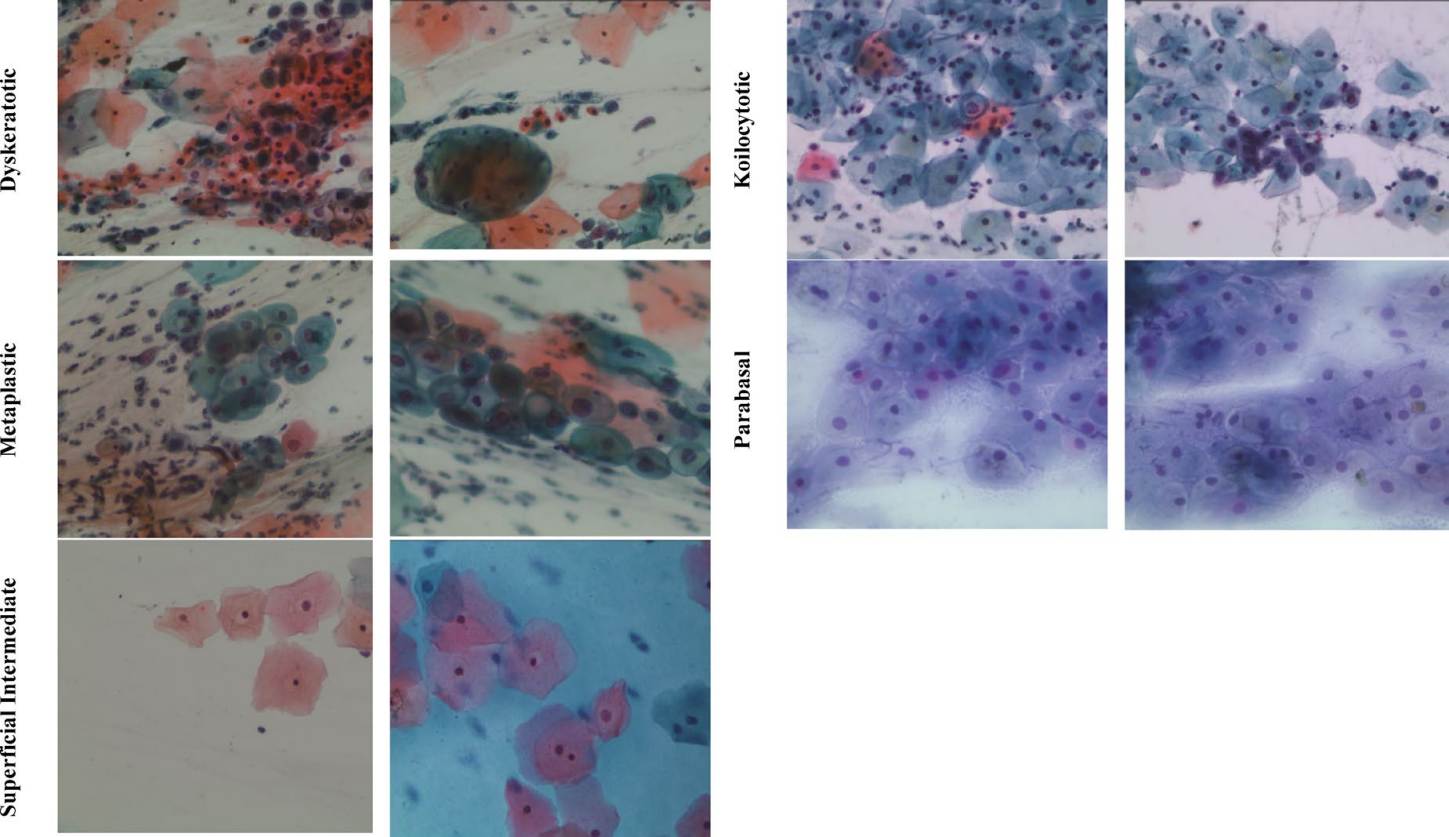
Cervical Cancer Subtypes-WSIs.

The SipakMed Database contains 4049 images featuring individual cells extracted from 966 group cell images obtained from whole slide images. These images are captured using a CCD camera mounted with an optical microscope. The cell images are sorted into five groups, including normal, abnormal, and benign. Normal cells are classified into “Superficial-Intermediate” and “Parabasal” categories, whereas abnormal cells, which are not malignant, are sorted into “Koilocytes” and “Dyskeratotic” groups. Additionally, a category for benign cells is called “Metaplastic” cells. The data set distribution is represented in Fig. 2. Figure 3 shows the subtypes of cervical cancer.

### Feature extraction using compound scaling convolutional neural network (CSCNN)

The histopathological images from the SipakMed dataset serve as input, where deep features are extracted using a Compound Scaling Convolutional Neural Network. Unlike existing deeper CNN architectures like DenseNet ResNet, our feature extraction model utilises a compound scaling method. The Compound scaling method scales the network’s depth $\:\widehat{\text{d}}$, width $\:\widehat{w}$, and resolution $\:\widehat{r}$ uniformly using a compound coefficient $\:d$. This ensures effective scalability and efficiency, calculated using the following Eqs. (1–3).

1 $$\:\text{D}\text{e}\text{p}\text{t}\text{h}:\:\:\widehat{\text{d}}={\alpha\:}^{\varnothing\:}$$

2 $$\:Width:\widehat{w}\:={\beta\:}^{\varnothing\:}$$

3 $$\:Resolution:\widehat{r}={\gamma\:}^{\varnothing\:}$$

where $\:\alpha\:$ to scale the depth (number of layers), $\:\beta\:\:$to scale width (number of channels per layer) and$\:\:\gamma\:$ to scale input resolution (height and width of input images). $\:\varnothing\:$ is the user-defined coefficient. In our model, the base network is scaled by compound coefficients with $\:\varnothing\:=1$ and a 1.2× deeper network, 1.1× wider channels, and 1.15× larger input resolution is achieved compared to the baseline CNNs.

**Fig. 4:**
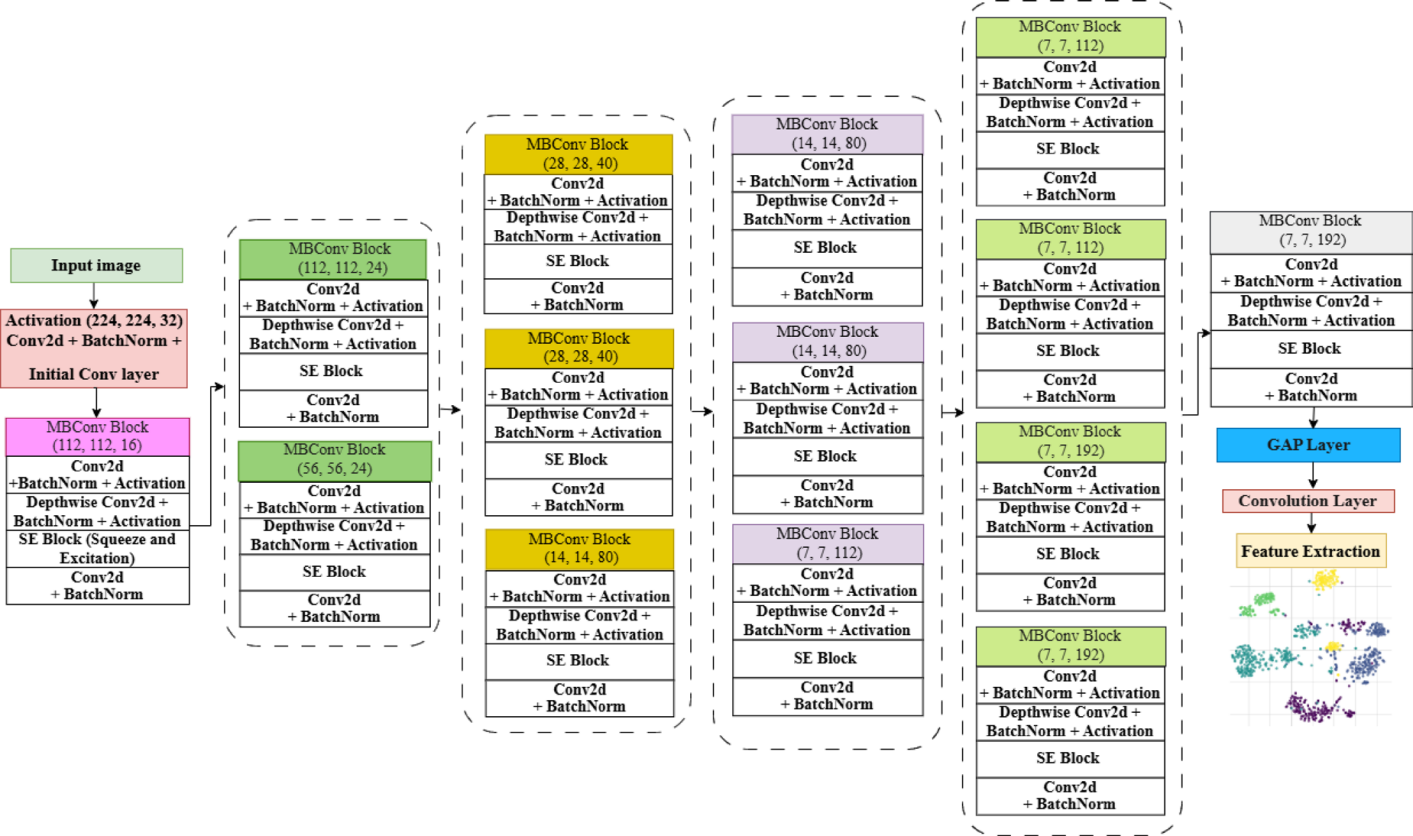
Feature extraction using compound scaling convolutional neural network.

The overall architecture of the CSCNN model is illustrated in Fig. 4. The architectural illustration shows a complete overview of the model, including the configuration of each layer, image size, stride and the most important functional components. CSCNN comprises 17 layers, with feature extraction eventually leading to the Global Average Pooling (GAP) layer. The GAP layer pools spatial information from the final feature maps into a low-dimensional feature vector well suited for the subsequent classification tasks. The model architecture initiates with a Conv2d layer, which preserves the spatial dimensions of the input feature maps by dynamically adjusting padding when stride $\:s$ = 1. The size of the output of the convolutional layer is defined by

4 $$\:{Output\:size}_{conv}=[size\:of\:the\:input/s]$$

where the padding size is calculated using$\:\:[f-1]/2$ based on the kernel size $\:f$, after convolution, the feature maps are normalised by Batch Normalisation (BN). BN normalises the activations for every mini batch, making the training process stable and faster. The convolution output is ensured to have a mean of 0 and a variance of 1, accompanied by learnable scaling and shifting operations. Equation (5) computes the batch normalisation’s ($\:\mathbf{Y}$) output.

5 $$\:\mathbf{Y}=\frac{\varvec{X}-\mu\:}{\sqrt{{\sigma\:}^{2}+\delta\:}}\:.\:\gamma\:+\beta\:$$

In this case, $\:\mathbf{X}$ is the input, while$\:\:\mu\:$ and $\:\sigma\:$ are the input’s mean and variance, respectively.$\:\:\gamma\:$ and $\:\beta\:$ stand for the learnable parameters. To prevent division by zero, $\:\delta\:$ is a small constant. The core building block of the CSCNN is a Mobile Inverted Bottleneck Convolution Block (MBConv block), which integrates five essential operations: depth-wise convolution, Projection, Squeeze and Excitation (SE) module, Expansion, and Swish Activation. The Expansion part verifies the expansion factor É. The input is extended using a $\:1\times\:1$ convolution if É is greater than 1. A Depth-wise Separable Convolution (DSC) is used in this stage, applying one convolutional filter per input channel with stride $\:s$ and kernel size $\:f$. DSC performs convolution independently over each input channel. The output feature map for depthwise convolution is mathematically represented as:

6 $$\:\mathbf{D}\left(\mathbf{X}\right)=\mathbf{X}*{\mathbf{f}}_{dw}$$

where $\:{\mathbf{f}}_{dw}$ is the depthwise kernel applied per channel. Equation (7) is utilised to determine the output size of this layer.

7 $$\:{Output\:size}_{dw}=\left[\frac{size\:of\:the\:input}{s}\right]$$.

Two fully connected layers (expansion and reduction) and a squeeze operation (global average pooling) are applied in the squeeze and excitation (SE) module to recalibrate channel-wise feature responses. The number of output channels after excitation is reduced by using a ratio factor $\:\omega\:$, given by:

8 $$\:{C}_{out}=\left[\frac{{c}_{in}}{\omega\:}\right]$$

Equation (8) can be used to define the output channels $\:{C}_{out}$ from SE, where $\:{c}_{in}$ denotes the number of input channels. The SE module recalibrates features using the sigmoid activation function. The output is reduced to the required number of channels by a final $\:1\times\:1$ pointwise convolution. Equation (9) defines the swish activation function, which is used.

9 $$\:Swish\:\left(\mathbf{X}\right)\:=\:\mathbf{X}.\:sigmoid\left(\mathbf{X}\right)\:$$.

The model analyses the input image using MBConv blocks and convolutional layers, gradually extracting higher-level features at each step. MBConv blocks collect more complex patterns and correlations in the data, while the first convolution layers capture low-level features. It efficiently extracts essential features using SE blocks, expands and projects convolutions, and depthwise separable convolutions.

The CSCNN model systematically improves feature representation in successive layers. Early convolutional layers handle low-level features (e.g., edges, textures), and high-level semantic patterns (e.g., cellular structures) are formed in deeper MBConv blocks. Combining depthwise separable convolutions, expansion-projection mechanisms, and SE blocks enables cost-effective feature extraction while ensuring parameter efficiency. Using compound scaling, CSCNN attains a high-resolution tradeoff between performance and model complexity and is thus suitable for histopathological image analysis.

### K-dimensional hypergraph neural network (kd-HGNN) with propagation for classification

The features extracted by CSCNN are subsequently processed by kd-HGNN. Relationships between sets of objects are represented by hypergraphs, which makes it possible to simulate complex interactions in a variety of fields, including computer science, data mining, social network analysis, and combinatorial optimisation. Unlike conventional graph-based models that rely on pairwise relationships, kd-HGNNs represent complex multi-node relationships, making them suitable for histopathological image classification. However, standard HGNN has limitations such as losing higher-order feature interactions and suboptimal graph construction techniques. To address these issues, we employ kd-HGNN, enhancing feature representation and classification accuracy.

$\:\varvec{G}=(\mathcal{V},\epsilon\:,\:\varvec{W})$ is the definition of a hypergraph, which consists of a vertex set $\:\mathcal{V}$, a hyperedge set $\:\epsilon\:$, and a hyperedge weight matrix $\:\varvec{W}$. A hypergraph $\:\varvec{G}$ can be represented as an $\:\left|V\right|\times\:\left|E\right|$ incidence matrix $\:\mathbf{H}$ whose entries are specified as

10 $$\:\mathbf{H}\left(v,e\right)=\left\{\begin{array}{c}1,\:if\:v\in\:V\\\:0,\:otherwise\end{array}\right.$$

Equation (10) specifies the incidence matrix $\:H$. The features of $\:N$ images in our classification task can be expressed as follows: $\:\mathbf{X}\:=[{\mathbf{x}}_{1},{\mathbf{x}}_{2}\:.\:.\:.\:,\:{\mathbf{x}}_{i}]$, where $\:{\mathbf{x}}_{\varvec{i}}$ is the feature vector of the $\:i$-th image. Each image is treated as a vertex in a hypergraph, and hyperedges are created between the vertices using feature vectors that have been retrieved. The k-dimensional-based hypergraph is constructed based on the distance between two features. The k-nearest neighbours of each feature vector are determined using Euclidean Distance as the distance function to generate hyperedges. Euclidean distance $\:d$ between two feature vectors $\:p$ and $\:q$ are represented as points in $\:n$-space, which can be computed by Eq. (11)

11 $$\:d(\mathbf{p}\:,\:\mathbf{q})\:=\:\sqrt{{\sum\:}_{i\:=\:1}^{n}({\mathbf{q}}_{i}\:-\:{\mathbf{p}}_{i}{)}^{2}}$$

where $\:{\mathbf{q}}_{i}$ and $\:{\mathbf{p}}_{i}$ are the Euclidean vectors, starting from the origin of the space (initial point). Each hyperedge weight is initialised to 1, represented by a diagonal matrix. Equation (12) specifies the diagonal matrix representation of weight.

12 $$\:\mathbf{W}\:=\:diag({w}_{1},{w}_{2},....,{w}_{n})$$

The hypergraph convolution can be formulated as,

13 $$\:{\mathbf{X}}^{l+1}\:=\sigma\:\left({{\mathbf{D}}_{\text{v}}}^{-\:\frac{1}{2}}\mathbf{H}\mathbf{W}{{\mathbf{D}}_{\text{e}}}^{-1}{\mathbf{H}}^{\text{T}}{{\mathbf{D}}_{\text{v}}}^{-\:\frac{1}{2}}{\mathbf{X}}^{\text{l}}{\varvec{\Theta\:}}^{\text{l}}\right)$$

Equation (13) is the mathematical representation of the hypergraph convolution operation, which propagates the features across the hypergraph structure. The node features at each layer get updated based on neighbouring node information through hyperedges. The symbols represent the diagonal matrices of vertex degrees and edge degrees. $\:{\mathbf{D}}_{v}$ and $\:{\mathbf{D}}_{e}$, respectively where $\:{\mathbf{D}}_{v}={\sum\:}_{e\in\:\epsilon\:}w\left(e\right)\mathbf{H}(v,e)$ and $\:{\mathbf{D}}_{e}={\sum\:}_{v\in\:\mathcal{V}}\mathbf{H}(v,e)$. $\:\varvec{\Theta\:}$ is the hypergraph propagation matrix and $\:\sigma\:$ is a non-linear activation function, RELU. The parameter to be learned during training is $\:\varvec{\Theta\:}\in\:{R}^{{C}_{1}\times\:{C}_{2}}$, where $\:{C}_{1}$ and $\:{C}_{2}$ are the feature dimensions. A three-layer hypergraph neural network has been employed. Batch normalisation is used, and the hidden layer’s channel count is 32. The cross-entropy loss function is minimised during training using the Adam optimiser, which has a learning rate 0.01.

**Fig. 5:**
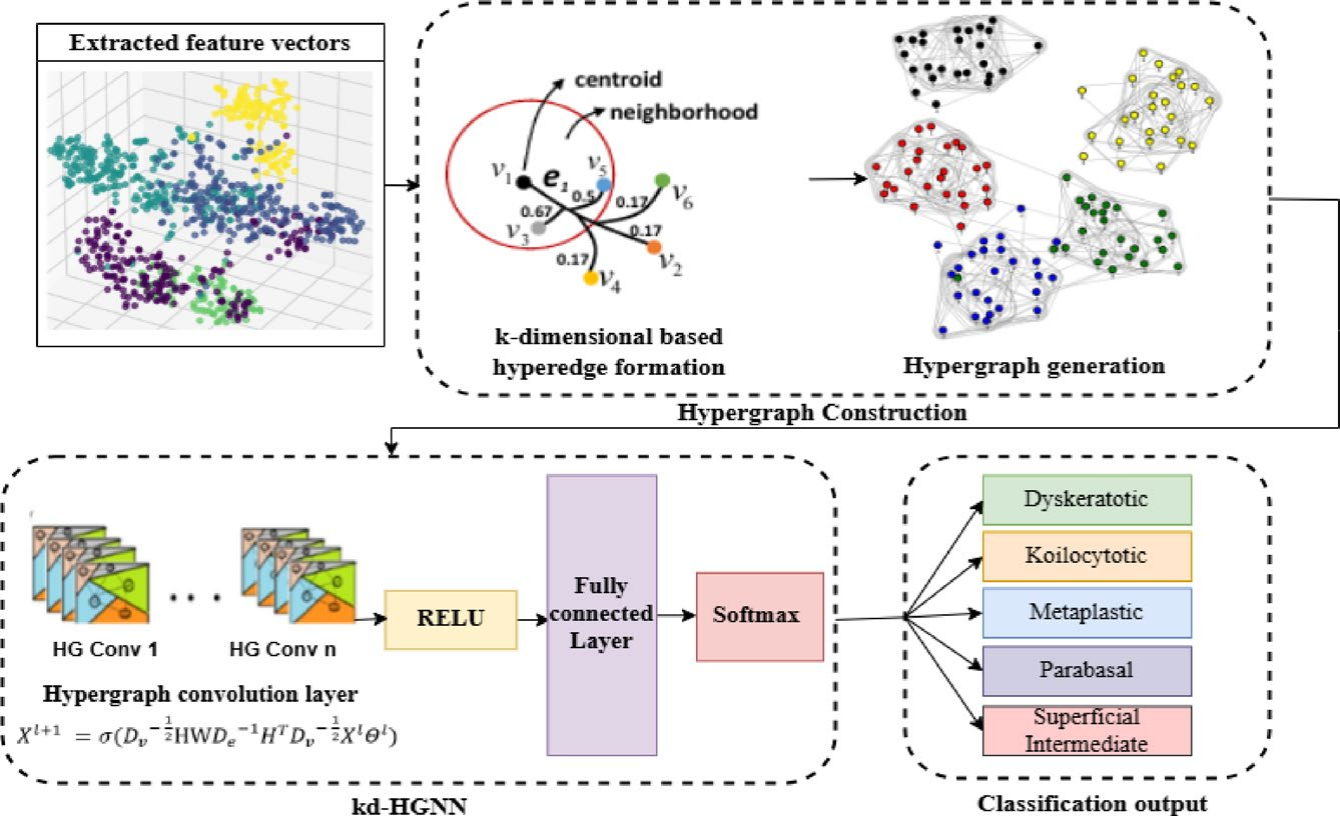
Overall working methodology of kd-HGNN.

Figure 5 illustrates the overall workflow of the classification model kd-HGNN. The proposed classification model, the kd-HGNN model, consists of two layers of hypergraph convolutional (HGConv). The input to the first HGConv layer is the 2304-dimensional feature vector obtained using the CS-CNN. Each HGConv layer is followed by the ReLu activation function and a dropout rate of 0.5 to prevent overfitting. The output of the HGConv layer is then passed through the fully connected layers, producing the final class probabilities via a SoftMax activation function. The model is trained using the Adam optimiser with a learning rate of 0.01 and weight decay of $\:5\times\:{10}^{-4}$. Training is done for 200 epochs and measured using typical classification metrics on the validation set, such as accuracy and F1-score. The model uses fixed hyperparameters such as learning rate, dropout rate, and number of neighbours $\:k$ in the hypergraph construction. However, it may exhibit sensitivity to hyperparameter tuning, and the performance could vary under different configurations.

## Experimental analysis and discussion

The proposed CSHG-CervixNet uses the Intel i9 processor 12th Generation workstation with 64GB RAM. A comprehensive evaluation of both five-fold cross-validation and hold-out validation was carried out. A comparative analysis between the conventional Graph Convolutional Network (GCN) and the proposed CSHG-CervixNet was conducted under both validation settings. Further, an ablation study was performed to analyse the effect of the hypergraph propagation mechanism, through the comparison of the performance of the model with and without the feature propagation module.

**Fig. 6:**
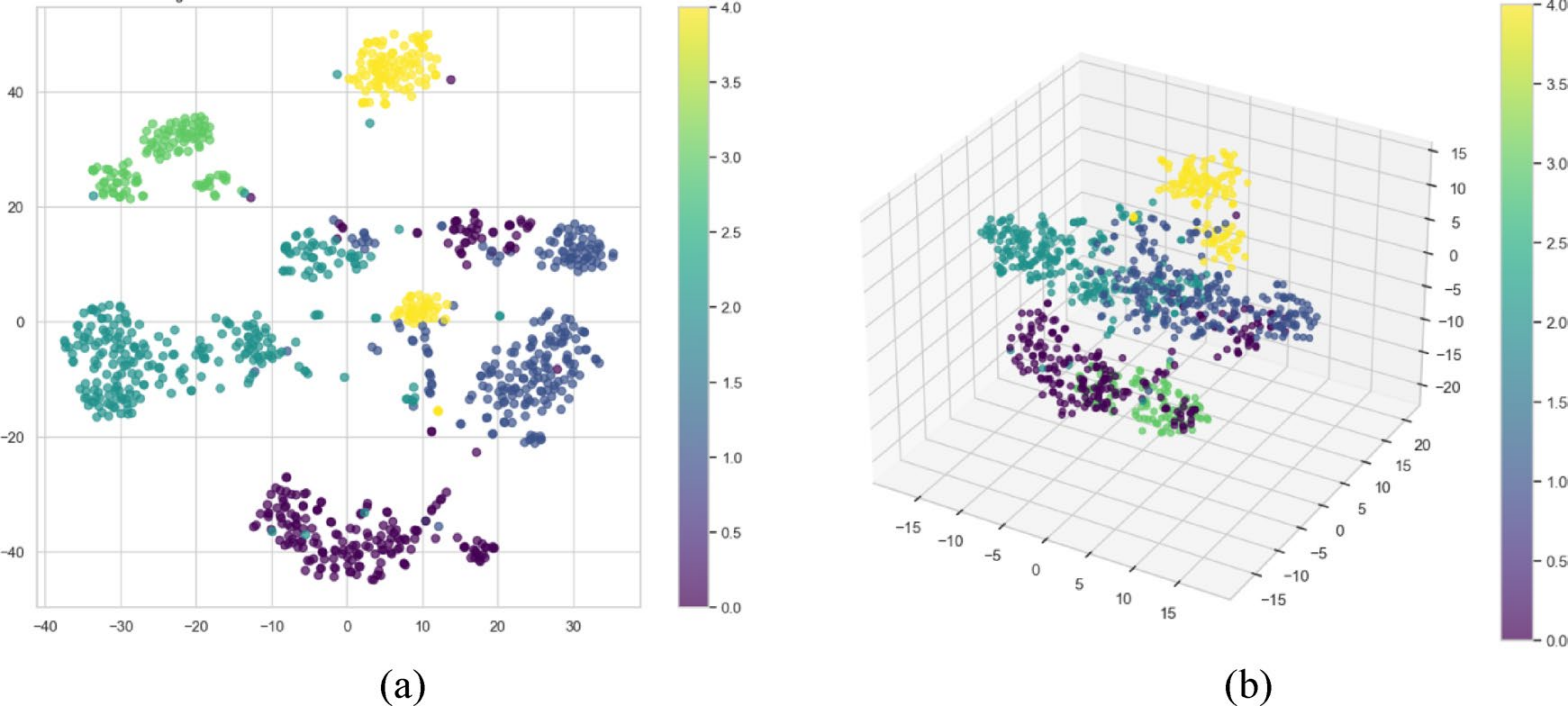
(**a**) t-SNE plot visualisation for the Raw Features (2D), (**b**) t-SNE plot visualisation for the Raw Features (3D).

The essential features are extracted using a Compound Scaling Convolutional Neural Network. t-SNE visualisation is employed to visualise the relationship between the features effectively. This t-SNE visualisation effectively explores the complex relationships and similarities among the various cervical cancer subtypes identified by the CSCNN. As can be seen from Fig. 6, the features are correctly classified and separated. A hypergraph is constructed from the extracted features from the CSCNN.

**Fig. 7:**
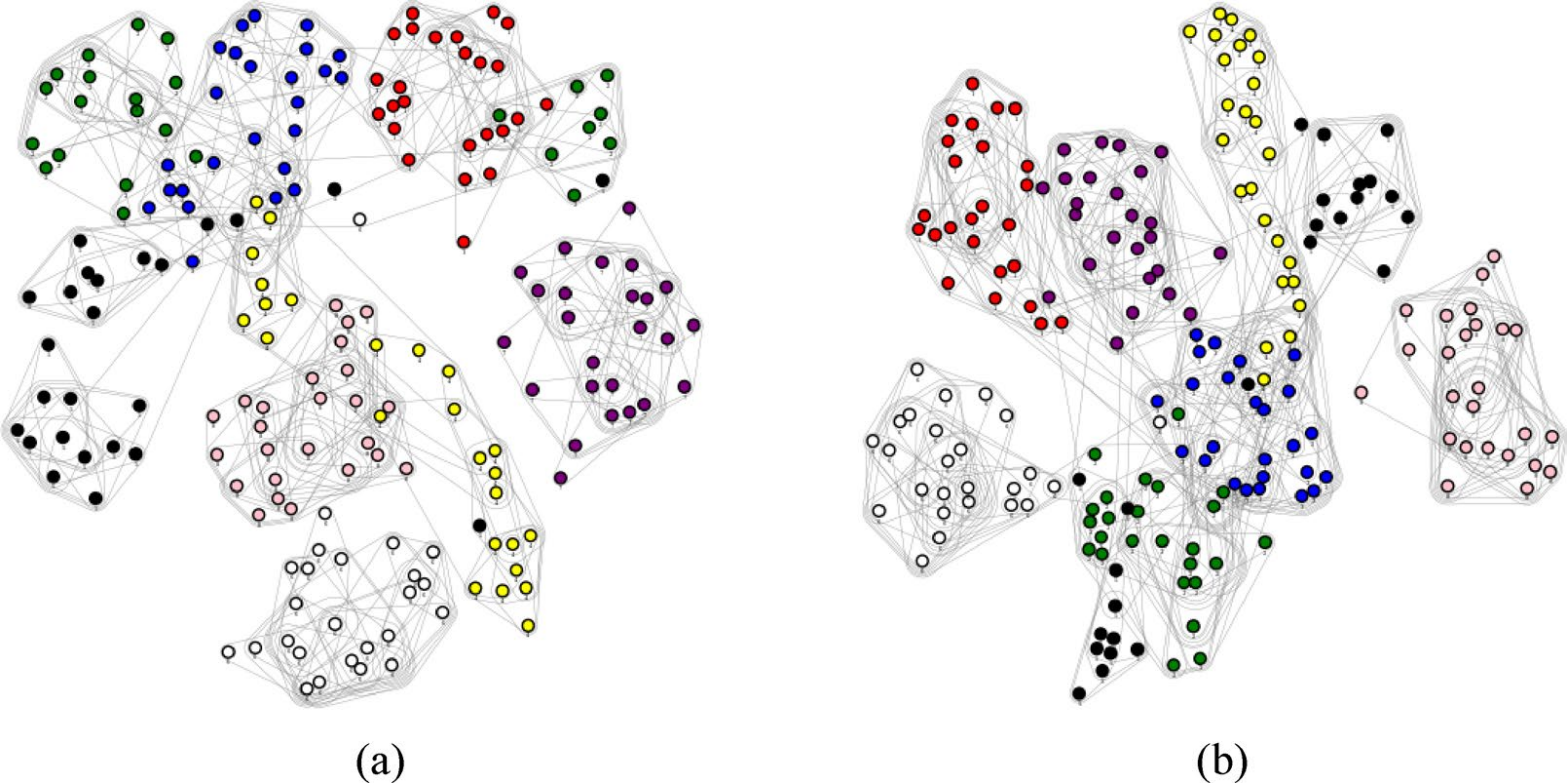
Sample hypergraph construction (**a**) k = 6: 25 images (**b**) k = 8: 25 images from the extracted images.

Figure 7 presents a sample hypergraph construction, where (a) represents $\:k=6$ with 25 images, and (b) represents $\:k=8\:$with 25 images, both derived from the extracted image dataset.

**Fig. 8:**
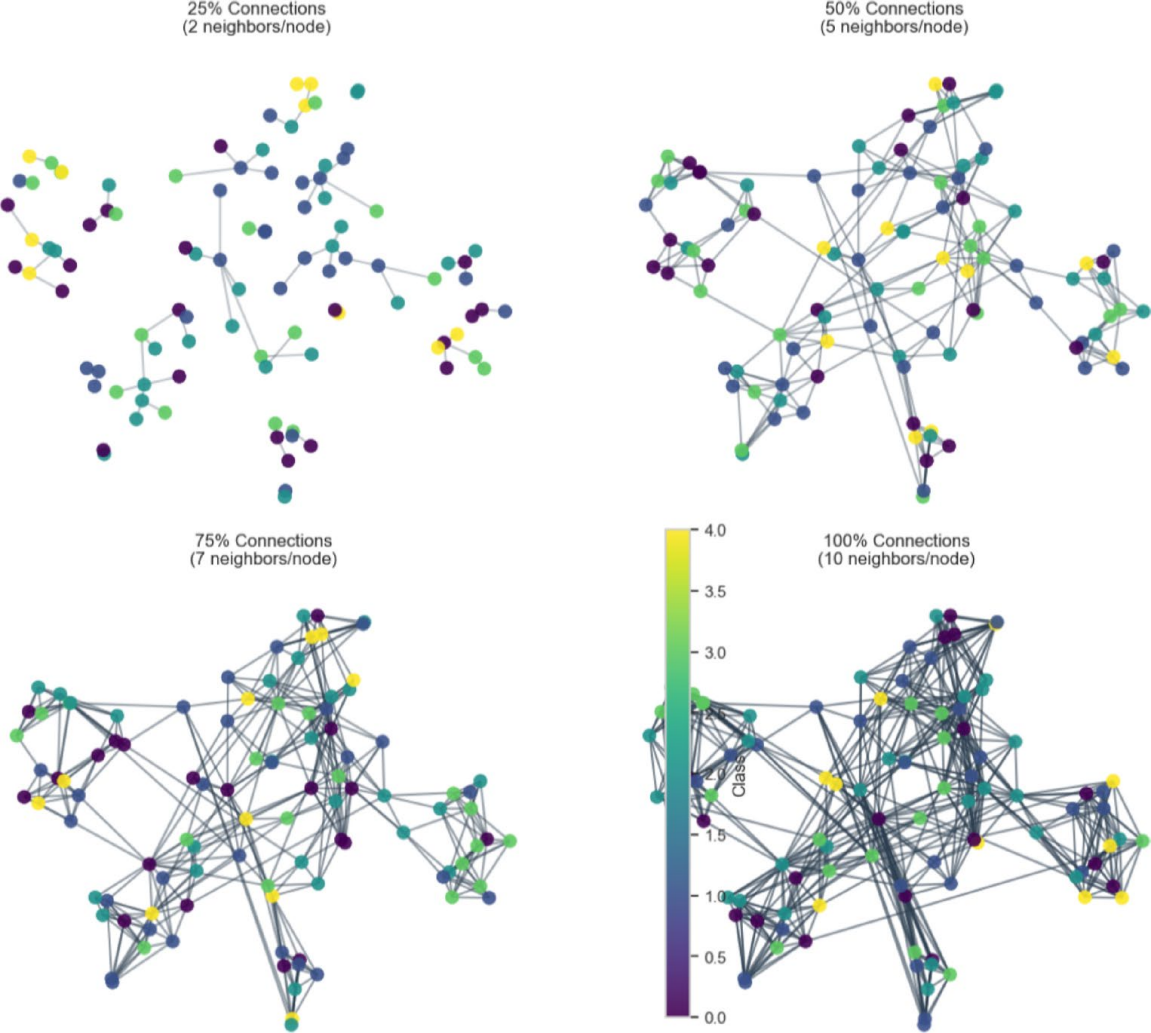
k-Nearest neighbor learning in the hypergraph.

Figure 8 shows the progressive construction of a k-Nearest Neighbours (KNN) graph with k = 10, showing increasing connectivity from 25% (2 neighbours per node) to 100% (10 neighbours per node).

**Fig. 9:**
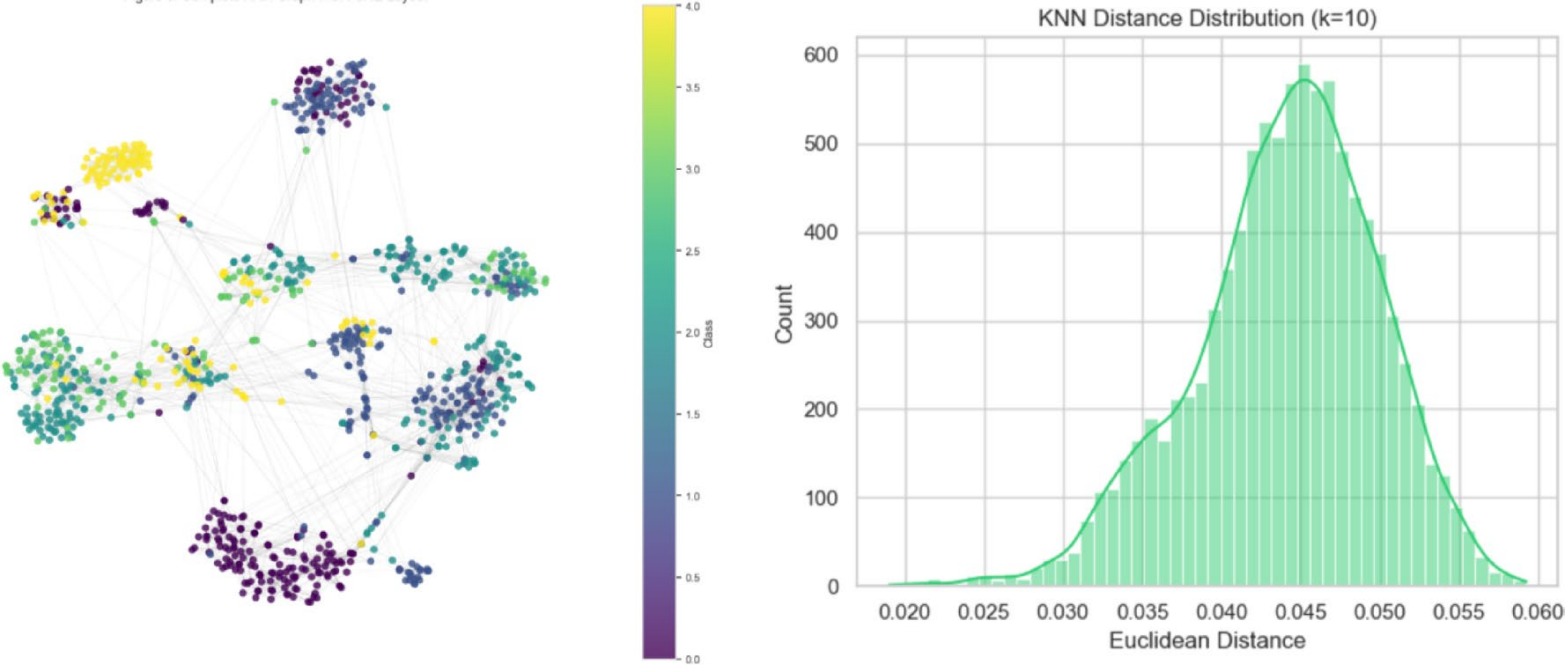
(**a**) Complete kNN graph with t-SNE visualisation, (**b**) K distance distribution (k = 10).

At 25% connectivity, where each node has only two neighbours, the network is sparse and limits node interactions. As connectivity increases to 50% with five neighbours per node, previously isolated clusters merge, forming a more structured and cohesive network. When the connectivity reaches 75% with seven neighbours per node, the network becomes significantly denser, improving inter-node relationships and strengthening overall cohesion. Ultimately, the graph creates a completely connected structure with 100% connectivity, where every node has ten neighbours, guaranteeing optimal interconnectivity. This makes it possible for information to spread throughout the network effectively. The colour bar in the visualisation represents different node classes or other pertinent metrics, which offer information about the graph’s categorisation or clustering patterns. Figure 9a shows the complete kNN graph with t-SNE visualisation. The KNN distance distribution for k = 10 shows most Euclidean distances clustering around 0.045 (Fig. 9b).

### Hold out validation results

For hold-out validation, a fixed partitioning approach was used to test the generalisation performance of the models. The dataset was split up into 70% training, 15% validation, and 15% test in a stratified manner to maintain class distribution and avoid sample overlap between subsets, thus preventing possible data leakage.

**Fig. 10:**
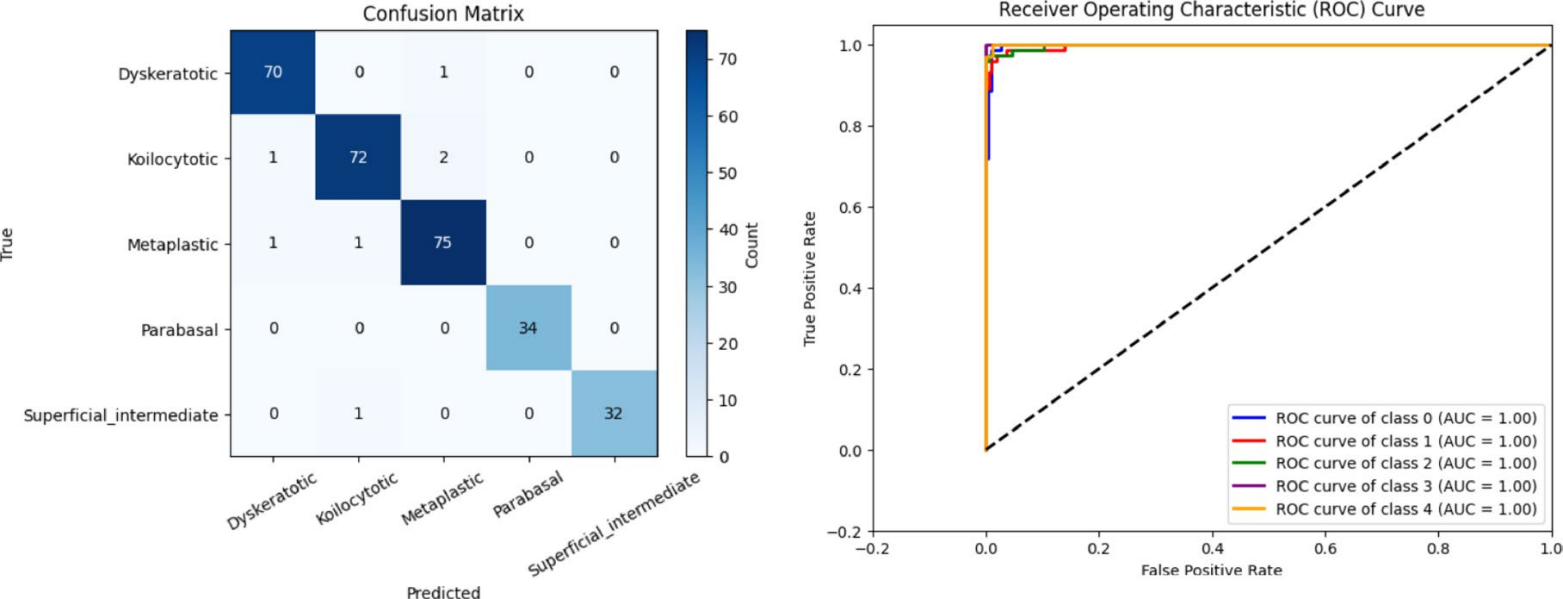
(**a**) Confusion matrix and (**b**) ROC curve for the convolutional GCN

Using hold-out validation, a comparative analysis has been conducted between conventional GCN and the proposed CSHG-CervixNet model. Figure 10a illustrates the confusion matrix of the GCN. Figure 10b depicts the ROC curve for the GCN.

**Fig. 11:**
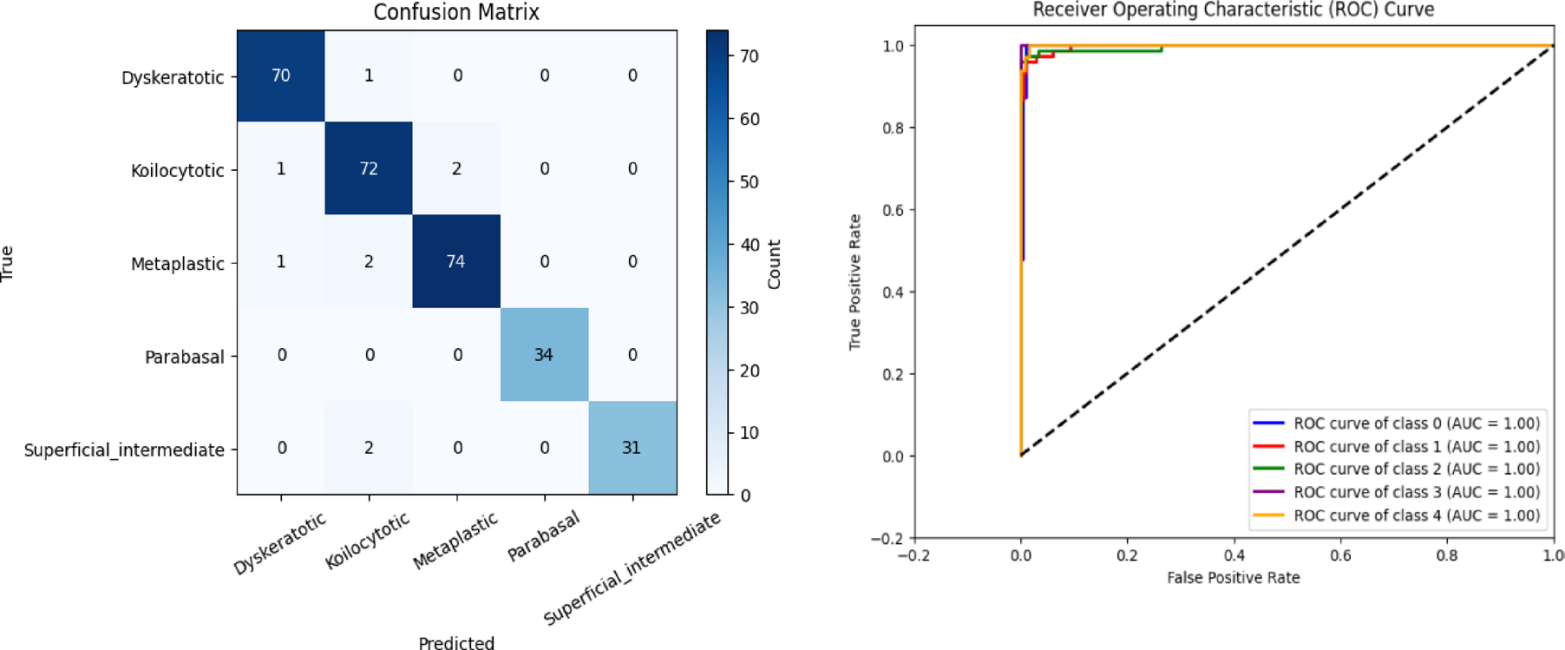
(**a**) Confusion matrix and (**b**) ROC Curve for the proposed CSHG-CervixNet.

Figure 11a shows the proposed model’s confusion matrix, clearly showing each class’s true positive, false positive, true negative, and false negative values. Compared to GCN, the confusion matrix reveals few misclassifications, highlighting the proposed model’s robustness in classifying cervical whole slide images. Table 2 shows the performance metrics of the proposed model evaluated by hold-out validation. The five classes’ accuracy, F1-score, recall, precision, and specificity are calculated separately. Figure 11b shows the ROC Curve for the proposed CSHG-CervixNet.

**Table 2:** The performance metrics of the proposed model CSHG-CervixNet using hold-out validation.

Performance metrics- hold out	Specificity	Precision	Recall	F1-score
Dyskeratotic	97.2	98.6	97.9	99.1
Koilocytotic	97.7	93.5	96	94.7
Metaplastic	99.1	97.3	96.1	96.7
Parabasal	100	100	100	100
Superficial_intermediate	100	93.9	96.9	100

The L2 regularisation technique was used to limit the model’s complexity and promote generalisation to avoid overfitting during training. This involves penalising high weight magnitudes, providing smoother optimisation and ensuring balanced feature propagation. Normalisation techniques were also incorporated to stabilise learning dynamics and reduce sensitivity to internal covariate shifts, mitigating the risk of overfitting.

### K-fold cross-validation results

**Fig. 12:**
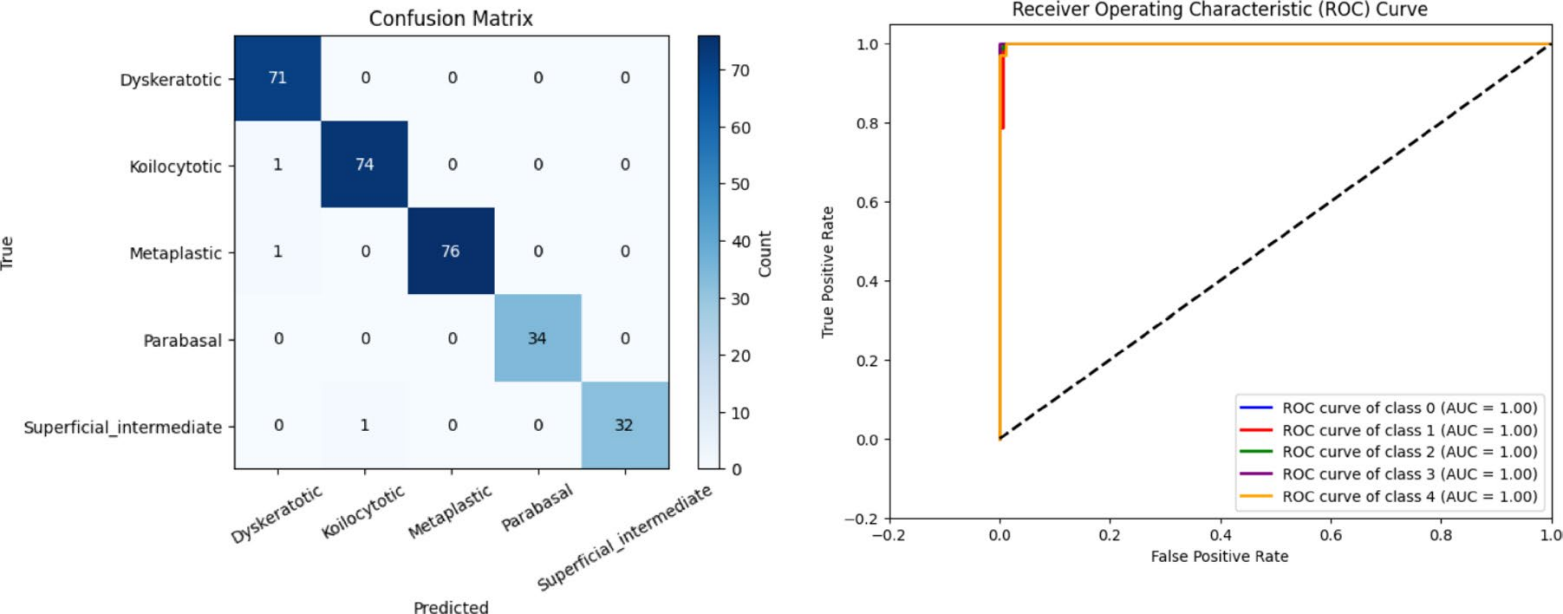
(**a**) Confusion matrix (**b**) ROC curve for conventional GCN.

To ensure the model’s robustness and generalizability, a fivefold cross-validation approach was utilised. First, the dataset was divided into five stratified folds to maintain the class distribution in each. One fold was kept aside as the test set in each iteration. The remaining four were divided into training and validation subsets, and 20% of the training fold was reserved for validation. The training set was used to train the model for 200 epochs in every fold, and the performance was tracked on the validation set. The parameters of the model that obtained the best validation score were saved and then tested on the respective test fold. Accuracy and F1-score performance metrics were calculated using a shared evaluator for every fold. This thorough cross-validation process guarantees an equitable evaluation of the model’s classifying ability for multiple data splits and reduces the overfitting potential inherent to hold-out validation. Figure 12a shows the confusion matrix of the conventional GCN, which demonstrates a moderate number of true positive detections, along with a significant number of false positives and negatives, especially in classes like Koilocytotic and Superficial_intermediate. Figure 12b shows the ROC curve of the GCN model. The overall accuracy attained by the GCN model is 98.96%.

**Fig. 13:**
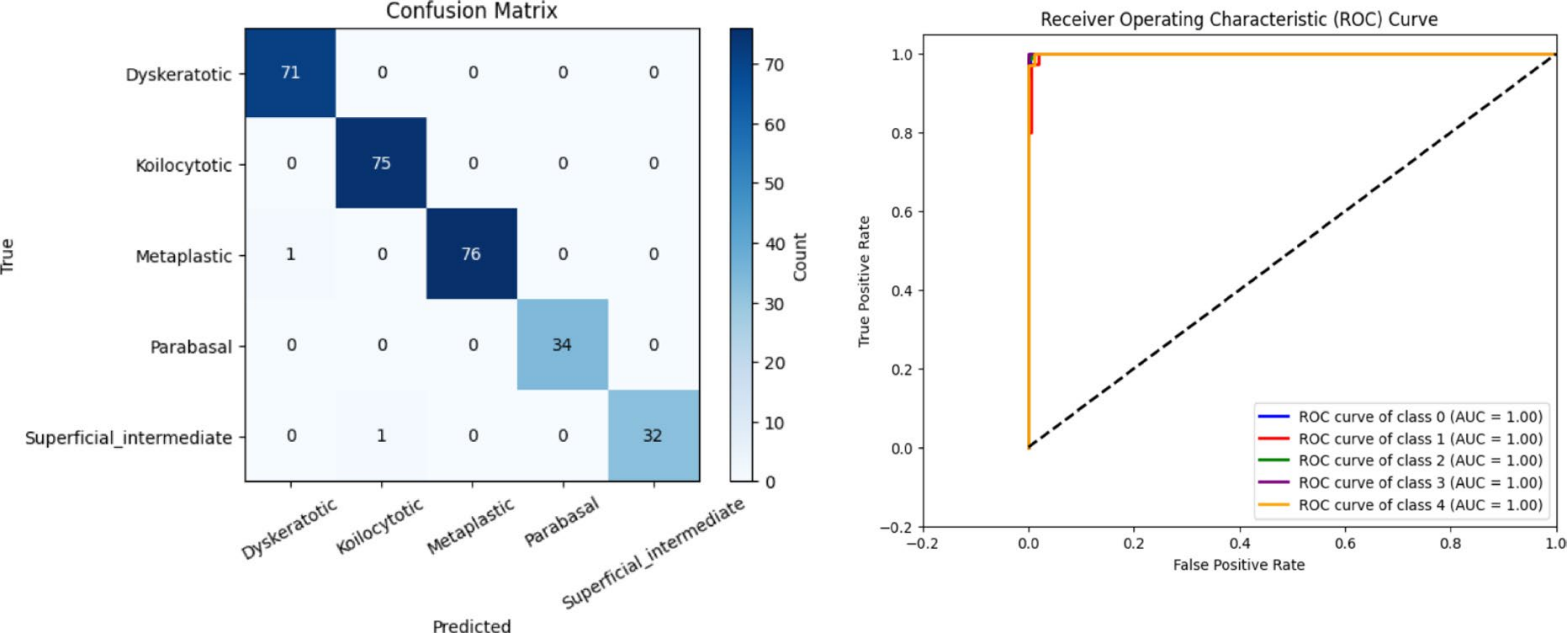
(**a**) Confusion matrix (**b**) ROC curve for the proposed CSHG-CervixNet.

Figure 13a shows the confusion matrix of the proposed CSHG-CervixNet, which yields considerably better true positive rates for all five classes. The reduced number of false positives and false negatives indicates a lower misclassification rate, highlighting the model’s improved accuracy and class discrimination capability. Figure 13b illustrates the ROC curve of the proposed model, highlighting its strong class-wise separability. The overall accuracy of CSHG-CervixNet is 99.31%, which outperforms the conventional GCN model.

The comparative analysis between the traditional GCN and the proposed CSHG-CervixNet model, as shown in Figs. 12 and 13, provides strong evidence of the superior classification performance achieved by the proposed CSHG-CervixNet. The class-wise performance metrics, such as Specificity, Precision, Recall, and F1-score for the proposed CSHG-CervixNet model, are tabulated in Table 3.

**Table 3:** Class-wise performance metrics of conventional CSHG-CervixNet using k-fold cross-validation.

Performance metrics- k-fold	Specificity	Precision	Recall	F1-score
Dyskeratotic	99.54	98.61	100	99.30
Koilocytotic	99.53	98.68	100	99.33
Metaplastic	100	100	98.70	99.34
Parabasal	100	100	100	100
Superficial_intermediate	100	100	96.96	98.46

The comparative analysis of the performance metrics GCN vs. CSHG-CervixNet with hold-out and k-fold cross-validation is tabulated in Table 4.

**Table 4:** Performance metrics of the CSHG-CervixNet with hold-out and k-fold cross-validation.

Model	Validation type	Accuracy (%)	Precision (%)	Recall (%)	F1-score (%)	Specificity (%)
GCN	Hold-out	97.58	97.67	97.59	97.57	99.12
GCN	5-Fold CV	98.96	98.95	98.93	98.62	99.99
CSHG-CervixNet	Hold-out	98.6	97.60	96.92	97.24	99.52
CSHG-CervixNet	5-Fold CV	99.31	98.97	99.38	99.34	99.77

The results show that CSHG-CervixNet outperforms the GCN model consistently in performance metrics such as accuracy, precision, recall, F1-score, and specificity. Interestingly, fivefold cross-validation produces better performance scores than hold-out validation for both models, suggesting better generalizability and robustness. More specifically, CSHG-CervixNet yields an accuracy of 99.31% and an F1-score of 99.34% through fivefold cross-validation, outperforming the baseline GCN by about 0.35% in accuracy and 0.72% in F1-score. In addition, the specificity levels above 99% in all experiments validate the model’s strong ability to identify negative samples correctly to avoid false positives. These results confirm the effectiveness of the CSHG-CervixNet model and highlight the advantage of using cross-validation in model testing.

### Ablation study

An ablation study was conducted with and without feature propagation to evaluate the effect of the hypergraph propagation mechanism. The CSHG-CervixNet model, when evaluated without propagation, recorded a precision of 98.72%, recall of 99.14%, and F1-score of 99.04% with a global accuracy of 98.97%. However, the model with propagation recorded better measures: precision of 98.97%, recall of 99.38%, F1-score of 99.34%, specificity of 99.77%, and accuracy of 99.31%. The propagation mechanism resulted in a significant decrease in misclassification and enhanced the consistency of class predictions. These results demonstrate the efficacy of integrating feature propagation into the hypergraph structure, augmenting the model’s ability to learn inter-class relationships and fine-grained feature variations in cervical WSIs.

**Table 5:** Performance metrics of CSHG-CervixNet with and without the hypergraph propagation mechanism.

Metric	Accuracy	Precision	Recall	F1-score	Specificity
Without propagation	98.96	98.72	99.14	99.04	99.72
With propagation	99.31	98.97	99.38	99.34	99.77

Table 5 shows that the CSHG-CervixNet model with a feature propagation mechanism outperformed the model without propagation across all key metrics. Table 6 compares the proposed model’s accuracy, F1-score, recall, and precision with the cutting-edge methods. The comparison table shows that our proposed CSHG-CervixNet model performs better than other state-of-the-art techniques.

**Table 6:** Comparison of the proposed CSHG-CervixNet with other state-of-the-art techniques.

Method/model	Accuracy	Precision	Recall	F1-score
BiNext-Cervix (CNN and Transformer-based modules)^5^	91.82	91.40	91.59	91.50
Progressive resizing + PCA^57^	98.47	98.72	98.97	99
UNet + GCN^62^	98.61%	97.33%	97.11%	97.56%
CCanNet (mobile transformer based model)^64^	98.58	98	100	99
Swin transformer^65^	95.50	-	-	-
Vision transformer^66^	97.247	97.253	97.247	97.239
Vision transformer ^63^	99.02	99.03	99.04	99.02
13 pre-trained deep CNN models (DenseNet201)^67^	87.02%	-	-	-
Densenet121^68^	86.14	86.90	85.58	86.24
HDFCN (Fine-tuned pre-trained models + Fully connected network)^60^	97.45	97.94	98.08	98.01
CervixFormer (Swin Transformer)^36^	91.56	91.12	91.32	91.22
VisionCervix (ViT + CNN)^45^	91.66	91.23	91.42	91.33
BiFormer (Bi-level Routing Attention based CNN)^69^	91.62	91.28	91.52	91.40
CNN based feature extraction + Cubic SVM classifier^70^	98.26	98.27	98.28	98.28
MLP^49^	96.54	96.87	96.15	96.93
CVM-Cervix (CNN, Visual Transformer + MLP)^20^	91.70	91.27	91.45	91.36
CytoBrain (Compact Visual Geometry Group (VGG))^55^	88.30	-	92.83	87.04
Graph convolutional network (GCN)^54^	98.37	99.80	99.60	99.80
ViT^71^	88.95	88.53	88.82	88.68
CSHG-CervixNet- compound scaling convolutional neural network + k-dimensional-based hypergraph convolutional neural network (ours)	99.31	98.97	99.38	99.34

The use of a hypergraph-based structure in our model CSHG-CervixNet facilitates the modelling of complex interdependencies between the deep image features. This approach enables robust performance across various evaluation metrics, accuracy (99.31%), precision (98.97%), recall (99.38%) and F1-score (99.34%). While models such as BiNext-Cervix and CervixFormer VisionCervix yield competitive results, they tend to exhibit certain limitations. Transformer-based models usually demand large-scale data to generalise well and are computationally expensive. Although hybrid models attempt to combine both strengths, they can become architecturally complex and costly, affecting scalability and interpretability. Our proposed hypergraph approach, by contrast, offers a distinct advantage in dealing with complex feature interactions, an aspect not directly handled by most of the traditional or hybrid models discussed. This makes the proposed approach a structurally unique alternative in the context of cervical cancer subtype classification. Even though the model attains better accuracy, the model’s performance is sensitive to hyperparameter settings, which require careful tuning for optimum results.

## Conclusion

A hybrid model for precisely classifying CC whole slide images is proposed. The model leverages a Compound Scaling Convolutional Neural Network to capture the images’ intricate morphological features. A robust k-dimensional-based Hypergraph Convolutional Neural Network is employed to further enhance the classification performance, which models the higher-order relationships between the feature vectors using the hyperedge construction based on neighbourhood similarity. The experimental analysis was conducted on the benchmark SipakMed dataset. The model achieved an overall classification accuracy of 99.31% and precision, recall, and F1-score values of 98.97%, 99.38%, and 99.34%, significantly outperforming the existing baseline techniques. The proposed model CSHG-CervixNet provides an efficient and accurate solution to automated cervical cancer subtype classification by addressing both feature representation and relational dependencies inherent in cytological data.

Although the model guarantees high classification performance, applying the framework to the multi-centre clinical database is challenging, as it involves variability in histopathological slide quality and patient demographics and integration with existing diagnostic workflows. Future studies should overcome these limitations by increasing model efficiency, hyperparameter search automation, and validating the methodology with diverse real-world clinical datasets. Model interpretability is one of the major considerations for clinical use. In future work, we will aim to integrate Explainable AI (XAI) methods to enable further insights into the model predictions.

## Data Availability

This work is based on the cervical cancer pap smear cytology images, and the SipakMed dataset is available in Kaggle. It is publicly available at https://www.kaggle.com/datasets/prahladmehandiratta/cervical-cancer-largest-dataset-SipakMed.
